# Supplementary material for: Rnd3 Is a Crucial Mediator of the Invasive Phenotype of Glioblastoma Cells Downstream of Receptor Tyrosine Kinase Signalling
Source: Cells. 2022 Nov 22;11(23):3716. doi: 10.3390/cells11233716 (PMC9741382; doi:10.3390/cells11233716)
Supplement: Supplementary file 1 [file cells-11-03716-s001.zip › cells-1985585-supplementary.pdf]

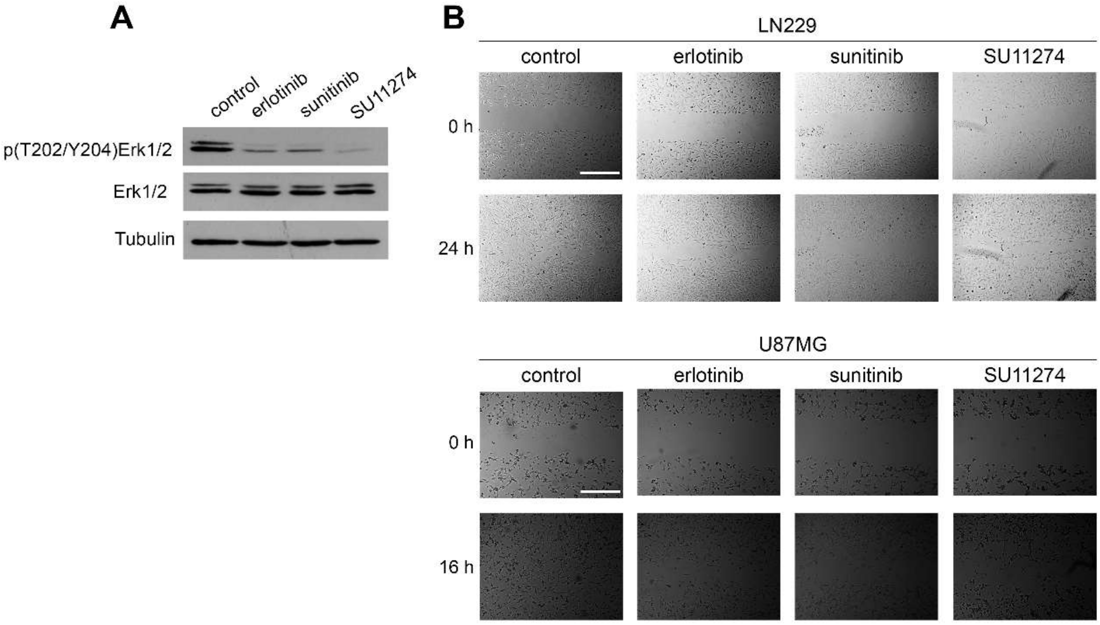

Figure S1. ***Receptor Tyrosine Kinase inhibition in GBM cells reduces cell motility and invasion.*** (a) U87MG were treated with 10 µM erlotinib, 2.5 µM sunitinib or 2.5 µM SU11274 for 16 hours, harvested and Phospho-Erk1/2 (Thr202/Tyr204) activation levels and total Erk1/2 expression levels were analysed by western blotting. (b) Representative phase-contrast micrographs of LN229 and U87MG cells treated either with vehicle (control) or with 10 µM erlotinib, 2.5 µM sunitinib or 2.5 µM SU11274 as indicated, at 0 h and 24 h (LN229) or 16 h (U87MG) after performing wound healing assays as described in Materials and Methods. Scale bar, 500 µm.

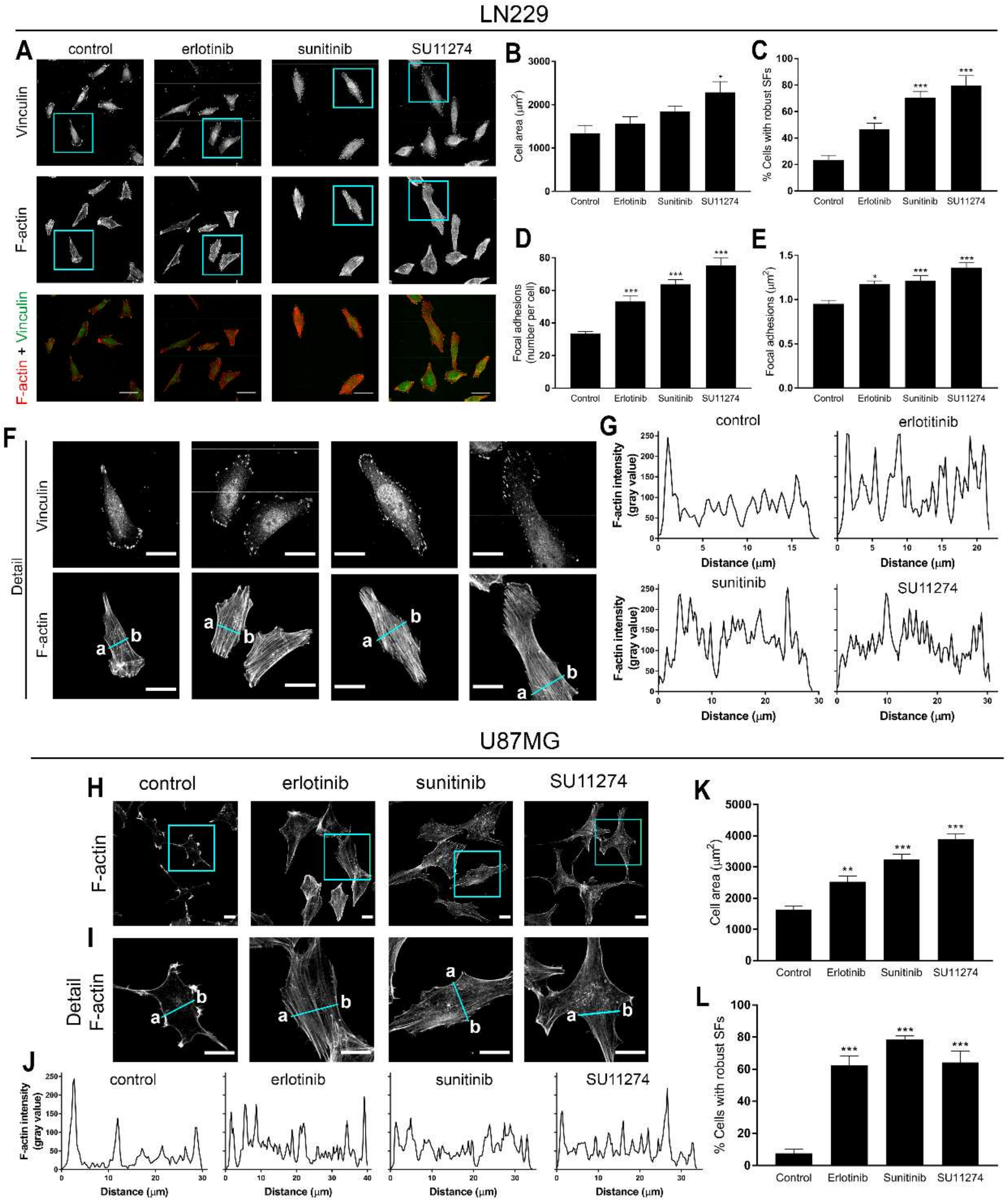

Figure S2. ***Receptor Tyrosine Kinase inhibition in GBM cells promotes actin cytoskeleton reorganization and focal adhesion assembly.*** (a) LN229 cells were grown on coverslips and were treated for 24 h with either vehicle (control) or with 10 µM erlotinib, 2.5 µM sunitinib or 2.5 µM SU11274 as indicated, fixed and stained with anti-vinculin antibody (Vinculin, green) and TRITC-labelled phalloidin (F-actin, red). Scale bar, 50 µm. (b) Quantification of cell area (µm²) and (c) percentage of cells with robust stress fibres (SFs) of LN229 cells from (a). Values represent the mean ± SEM from three independent experiments. Total number of cells analysed: n=192 control, n=297 erlotinib, n=121 sunitinib, n=138 SU11274. Statistical significance was assessed by one-way analysis of variance (ANOVA) with Dunnett's multiple comparisons test. (d) Quantification of focal adhesion number per cell and (e) average focal adhesion area per cell of LN229 cells from (b). Values represent the mean ± SEM from n=60 control, n=46 erlotinib, n=31 sunitinib and n=47 SU11274 cells. Statistical significance was assessed using a Kruskal-Wallis nonparametric test with Dunn's multiple comparisons test. (f) Magnified images from the boxed regions in (a); scale bar, 25 µm. (g) Intensity profiles of F-actin from the blue lines drawn in (f) from a to b. (h) U87MG cells were grown on coverslips and were treated for 16 h with either vehicle (control) or with 10 µM erlotinib, 2.5 µM sunitinib or 2.5 µM

SU11274 as indicated, fixed and stained and TRITC-labelled phalloidin (F-actin). Scale bar, 25 μm. (i) Quantification of cell area (μm^2) and (j) percentage of cells with robust stress fibres (SFs) of U87MG cells from (h). Values represent the mean ± SEM from five independent experiments. Statistical significance was assessed by one-way analysis of variance (ANOVA) with Dunnett's multiple comparisons test. Total number of cells analysed: n=271 control, n=262 erlotinib, n=177 sunitinib, n=192 SU11274. (k) Magnified images from the boxed regions in (h); scale bar, 25 μm. (l) Intensity profiles of F-actin from the blue lines drawn in (k) from a to b. Statistical significance is indicated as follows: *p<0.05, **p<0.01, ***p<0.001.

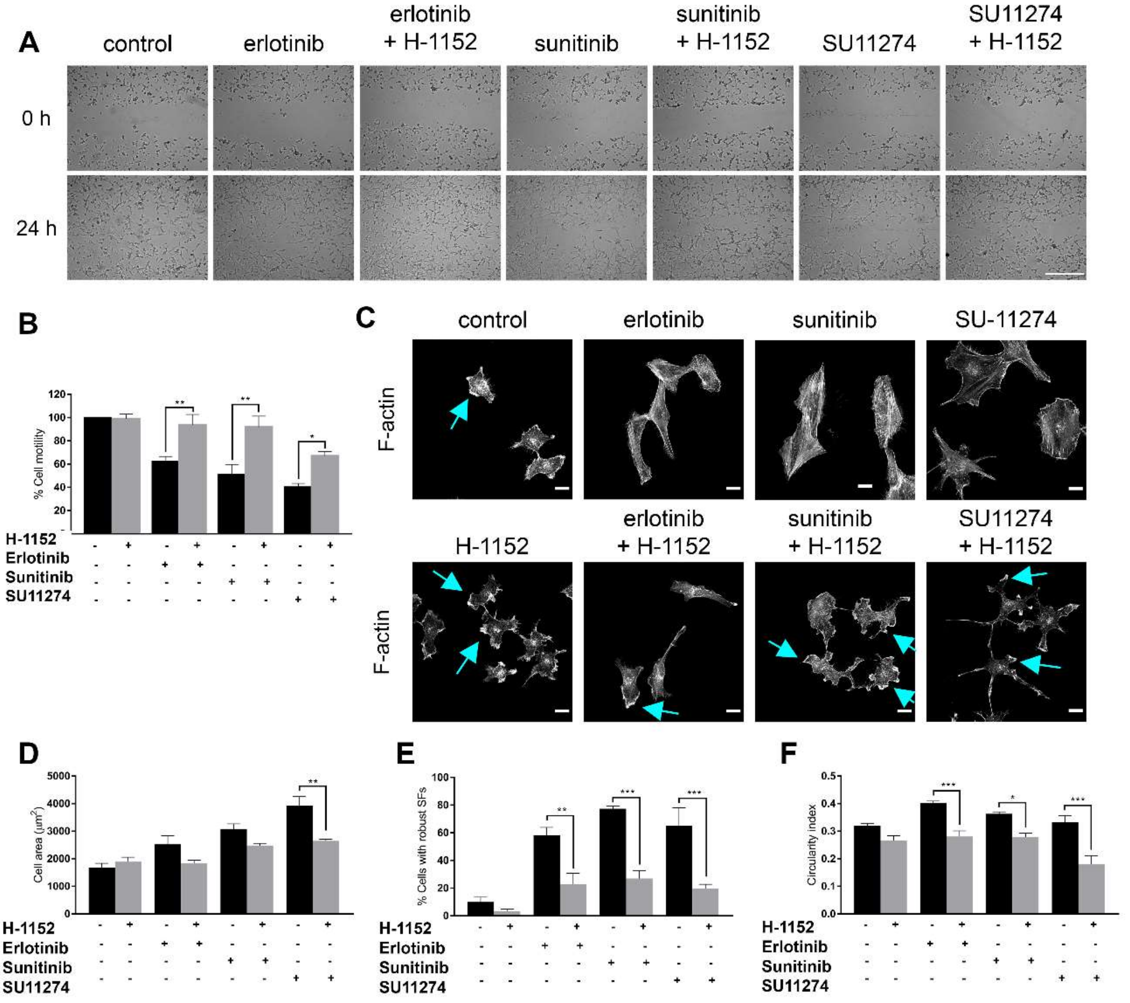

Figure S3. ***Receptor Tyrosine Kinase inhibition in GBM cells regulates cell motility and actin cytoskeleton reorganization through Rho GTPase modulation.*** (a) Representative phase-contrast micrographs of U87MG cells treated with vehicle (control) or with 10 μM erlotinib, 2.5 μM sunitinib or 2.5 μM SU11274 alone or in the presence of 0.5 μM H-1152, at 0 h (upper panel) or 16 h after (lower panel) performing wound healing assays as described in Materials and Methods. (b) Representation of the mean ± SEM rate of motility of U87MG cells from three independent experiments, expressed as the percentage of cell motility relative to Control cells. Statistical significance was assessed by one-way analysis of variance (ANOVA) with Dunnett's multiple comparisons test. (c) U87MG cells were grown on coverslips and were treated for 16 h with vehicle (control) or with 10 μM erlotinib, 2.5 μM sunitinib or 2.5 μM SU11274 alone (upper panel) or in the presence of 0.5 μM H-1152 (lower panel), fixed and stained with TRITC-labelled phalloidin (F-actin). Blue arrows, lamellipodia; scale bar, 25 μm. (d) Quantification of cell area (μm^2), (e) percentage of cells with robust stress fibres (SFs) an (f) circularity index of p01 cells from (c). Values represent the mean ± SEM from three independent experiments. Total number of cells analysed: n=157 control, n=156 H-1152, n=167 erlotinib, n=147 erlotinib+H-1152, n=122 sunitinib, n=124 sunitinib+H-1152, n=143 SU11274, n=140 SU11274+H-1152.

Statistical significance was assessed by one-way analysis of variance (ANOVA) with Dunnett's multiple comparisons test. Statistical significance is indicated as follows: *p<0.05, **p<0.01, ***p<0.001.

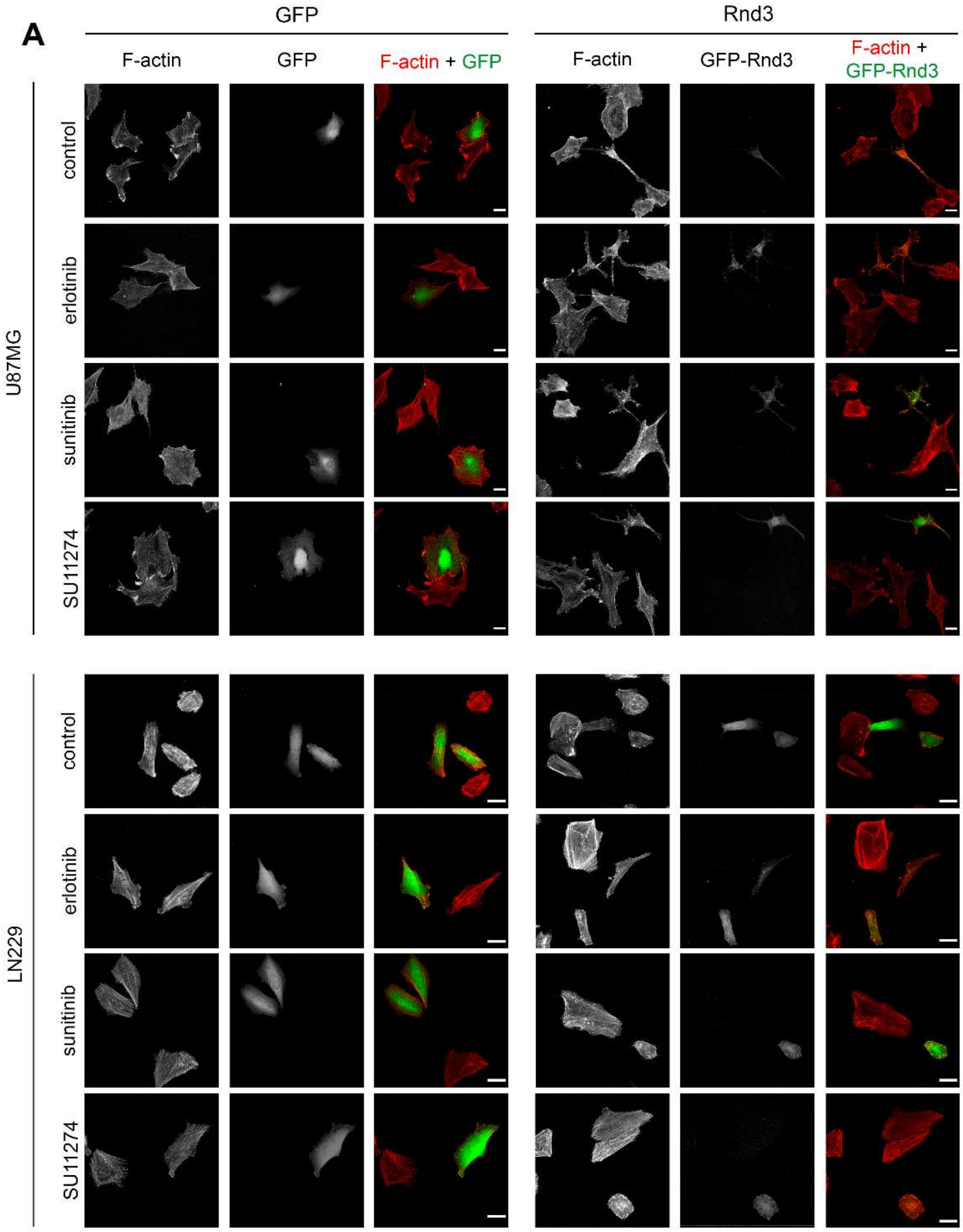

Figure S4. Rnd3 is a downstream target of RTK signalling that participates in actin organization. (a) U87MG and LN229 cells were transfected with Rnd3-GFP or GFP encoding plasmids, treated for 16 hours (U87MG) or 24 hours (LN229) with 10 µM erlotinib, 2.5 µM sunitinib or 2.5 µM SU11274, fixed and stained with TRITC-labelled phalloidin (F-actin). Scale bar, 25 µm.

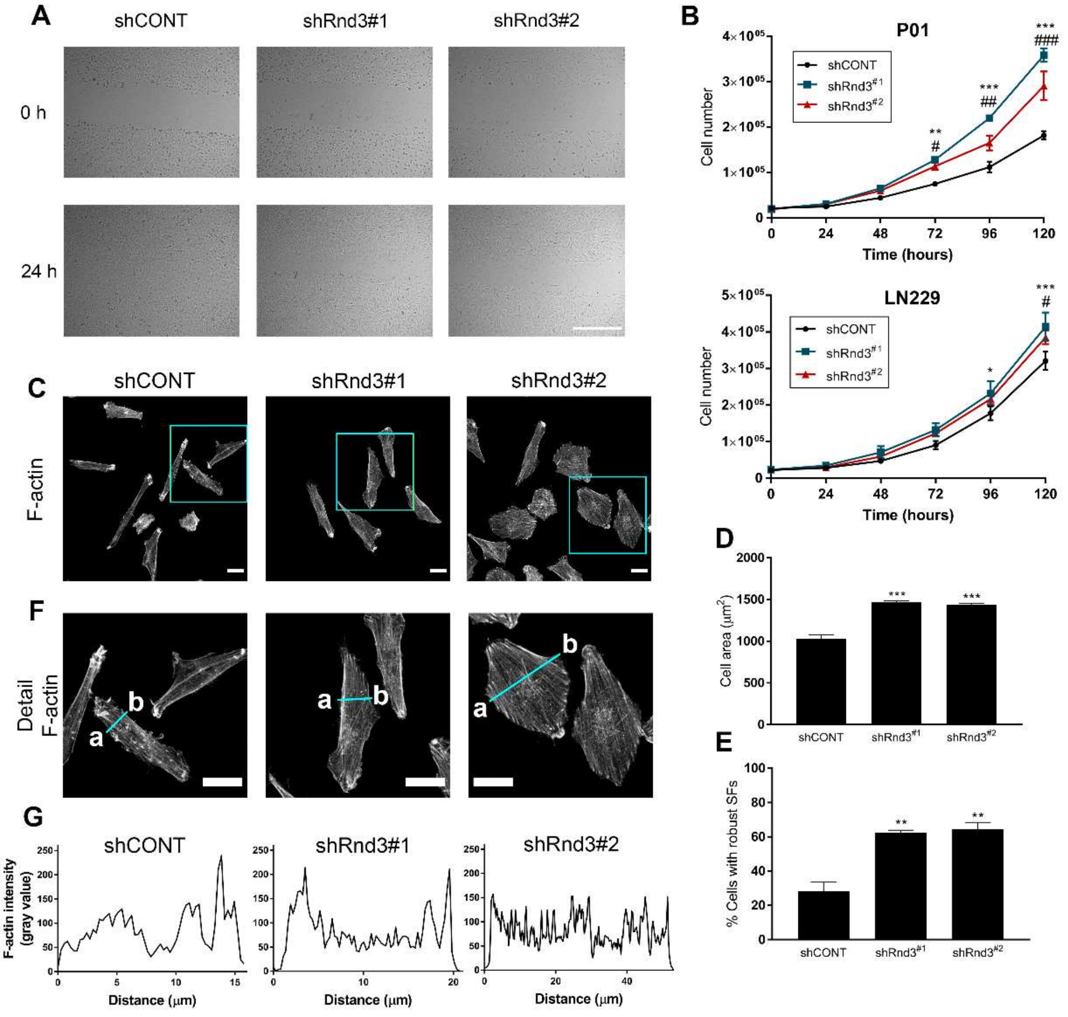

Figure S5. Rnd3 is a crucial mediator of cell motility and cytoskeleton dynamics in GBM cells. (a) Representative phase-contrast micrographs of control (shCONT) and Rnd3-depleted (shRnd3) LN229 cells at 0 h (upper panel) and 24 h after (lower panel) performing wound healing assays as described in Materials and Methods. Scale bar, 500 µm. (b) Growth of P01 and LN229 control (shCONT) and Rnd3-depleted (shRnd3) cells over a 120-hour period. Data points represent the mean ± SEM from three independent experiments. Statistical significance was assessed using a repeated measures two-way analysis of variance (ANOVA) with Dunnett's multiple comparisons test. Data points represent the mean ± SEM from three independent experiments. (c) LN229 control (shCONT) and Rnd3-depleted (shRnd3) cells were grown on coverslips, fixed and stained TRITC-labelled phalloidin (F-actin). Scale bar, 25 µm. (d) Quantification of cell area (μm^2) and (e) percentage of cells with robust stress fibres (SFs) of LN229 cells from (c). Values represent the mean ± SEM from three independent experiments. Total number of cells analysed: n=141 shCONT, n=161 shRnd3$^{\#1}$, n=172 shRnd3$^{\#2}$. Statistical significance was assessed by one-way analysis of variance (ANOVA) with Dunnett's multiple comparisons test. (f) Magnified images from the boxed regions in (c); scale bar, 25 µm. (g) Intensity profiles of F-actin from the blue lines drawn in (f) from a to b. Statistical significance is indicated as follows: $^{*}p<0.05$, $^{**}p<0.01$, $^{***}p<0.001$. In (b), (*) indicates differences between shCONT vs shRnd3$^{\#1}$, (#) indicates differences between shCONT and shRnd3$^{\#2}$.
